# Lower serum 25(OH)D levels associated with higher risk of COVID-19 infection in U.S. Black women

**DOI:** 10.1371/journal.pone.0255132

**Published:** 2021-07-27

**Authors:** Yvette C. Cozier, Nelsy Castro-Webb, Natasha S. Hochberg, Lynn Rosenberg, Michelle A. Albert, Julie R. Palmer

**Affiliations:** 1 Slone Epidemiology Center at Boston University, Boston, Massachusetts, United States of America; 2 Section of Infectious Diseases, Department of Medicine, Boston University School of Medicine, Boston, Massachusetts, United States of America; 3 Center for Infectious Diseases, Boston Medical Center, Boston, Massachusetts, United States of America; 4 Division of Cardiology, Department of Medicine, University of California San Francisco School of Medicine, Center for the Study of Adversity and Cardiovascular Disease (NURTURE Center), San Francisco, California, United States of America; University of Insubria, ITALY

## Abstract

**Objective:**

Limited evidence suggests that higher levels of serum vitamin D (25(OH)D) protect against SARS-CoV-2 virus (COVID-19) infection. Black women commonly experience 25(OH)D insufficiency and are overrepresented among COVID-19 cases. We conducted a prospective analysis of serum 25(OH)D levels in relation to COVID-19 infection among participants in the Black Women’s Health Study.

**Methods:**

Since 1995, the Black Women’s Health Study has followed 59,000 U.S. Black women through biennial mailed or online questionnaires. Over 13,000 study participants provided a blood sample in 2013–2017. 25(OH)D assays were performed in a certified national laboratory shortly after collection of the samples. In 2020, participants who had completed the online version of the 2019 biennial health questionnaire were invited to complete a supplemental online questionnaire assessing their experiences related to the COVID-19 pandemic, including whether they had been tested for COVID-19 infection and the result of the test. We used logistic regression analysis to estimate odds ratios (OR) and 95% confidence intervals (CI) for the association of 25(OH)D level with COVID-19 positivity, adjusting for age, number of people living in the household, neighborhood socioeconomic status, and other potential confounders.

**Results:**

Among 5,081 eligible participants whose blood sample had been assayed for 25(OH)D, 1,974 reported having had a COVID-19 test in 2020. Relative to women with 25(OH)D levels of 30 ng/mL (75 nmol/l) or more, multivariable-adjusted ORs for COVID-19 infection in women with levels of 20–29 ng/mL (50–72.5 nmol/l) and <20 ng/mL (<50 nmol/l) were, respectively, 1.48 (95% CI 0.95–2.30) and 1.69 (95% CI 1.04–2.72) (p trend 0.02).

**Conclusion:**

The present results suggest that U.S. Black women with lower levels of 25(OH)D are at increased risk of infection with COVID-19. Further work is needed to confirm these findings and determine the optimal level of 25(OH)D for a beneficial effect.

## Introduction

The global pandemic caused by SARS-CoV-2 virus (COVID-19) infection has touched people in all walks of life since its emergence in China in December 2019 [1, 2]. In the U.S. and worldwide, millions of people became infected with the virus and died in the first year alone [3, 4]. The spread of COVID-19 infection has been especially high in Black Americans, who tend to live in dense, low-resource communities and work in occupations in which they are more likely to be exposed to the virus [5–8]. In addition, Black women are more likely to be the economic breadwinners of their households and serve as caregivers within multi-generational households, thereby increasing exposure to the virus outside and inside the household [5, 9–11].

Several recent studies have examined the role of vitamin D levels in relation to COVID-19 infection, with inconsistent results [12–16]. Although some [12–15] found an increased occurrence of infection in those with lower 25(OH)D levels, data from the UK Biobank indicated similar 25(OH)D levels among those who tested positive to COVID-19 and those who did not [16]. The largest U.S. study, based on results from a national laboratory, provides the strongest evidence of an association between lower 25(OH)D level and increased risk of COVID in U.S. populations, but was limited by incomplete data on race/ethnicity [12].

Vitamin D is a hormone found in nature that regulates mineral and skeletal homeostasis and modulates the innate immune system [17]. In humans, vitamin D is obtained from diet as plant-, yeast-, and mushroom-derived ergocalciferol (vitamin D2) and cholecalciferol (vitamin D3), or through dietary supplements [18]. Another major source is through exposure to ultraviolet-B radiation, which is involved in converting 7-dehyrocholesterol to vitamin D3 [25(OH)D] by the liver [19, 20].

U.S. Blacks commonly experience (25(OH)D) insufficiency (20 to 29 ng/mL; 50–72.5 nmol/l) or deficiency (<20 ng/mL; <50 nmol/l), largely because darker skin pigmentation [17, 21] results in lower penetration of sunlight and subsequent production of vitamin D_3_ [22, 23]. Once infected with COVID-19, Black women are particularly vulnerable to developing severe disease, in part due to high levels of predisposing conditions such as hypertension, type 2 diabetes, and obesity [24]. Given the overrepresentation of Black women among COVID-19 cases and deaths [25] and the high levels of 25(OH)D insufficiency among Black women [21], it is particularly important to evaluate the relation of serum 25(OH)D concentrations to risk of COVID-19 infection in this population group. We did so among participants in the Black Women’s Health Study (BWHS), a large national follow-up study of U.S. Black women, with careful control for potential confounding by the higher levels of exposure to COVID-19 experienced by U.S. Blacks.

## Materials and methods

### The Black Women’s Health Study

In 1995, Black women ages 21–69 years (median 38 years) from the continental U.S. enrolled in the BWHS by completing a 14-page health questionnaire; those found to have valid addresses a year later (N = 59,000) have been followed by biennial health questionnaires, on which participants provide demographic, medical, and lifestyle information [26, 27]. The questionnaires used are available at www.bu.edu/bwhs. Yearly linkage with the National Death Index is used to identify deaths. Follow-up has been successful for >80% of potential person-years through the end of the 2019. The Institutional Review Board of Boston University Medical Center approved the study. Participants indicated their consent by completing the questionnaires. In addition, participants who provided a blood sample provided a signed informed consent to allow the samples to be used for future health research.

### BWHS COVID-19 study

In August 2020, we invited the approximately 17,000 BWHS participants who completed the online version of the 2019 biennial questionnaire to complete a supplemental online COVID-19 questionnaire (available at www.bu.edu/bwhs). The questionnaire assessed participants’ experiences related to COVID-19, diagnostic testing, hospitalization, and the occurrence of selected health conditions after infection (e.g., heart attack, stroke, peripheral neuropathy). Women were specifically asked whether they had ever had a COVID-19 test, and the results of the test (positive, negative, unsure). They were asked about living arrangements, including whether they live alone or live with a partner/spouse, children, parents, other relatives, friends or roommates, and how many people in all. Another question asked participants to respond yes or no to the statement “I had to continue work at my workplace even though it was unsafe.” Finally, for those who reported COVID-19 infection, we asked whether their health had returned to the usual level it had been before they had become infected. The supplemental questionnaire was completed by 10,268 women from August 1 through December 1, 2020.

### Vitamin D (25(OH)D)

Blood specimens were collected from 13,030 BWHS participants from 2013 through 2017. Samples were collected and tested at regional laboratories of Quest Diagnostics (Madison, New Jersey; www.QuestDiagnostics.com), a national clinical laboratory certified under the Clinical Laboratory Improvement Amendments of 1988 [28]. Vitamin D assays were performed within 72 hours of blood collection using liquid chromatography–tandem mass spectrometry, a sensitive and specific method for measuring serum levels of circulating 25(OH)D. National Institute of Standards and Technology Standard Reference Material for 25(OH)D in human serum was used for quality control. Participants who provided samples gave written informed consent to use the blood samples for health-related research for the entirety of the BWHS study period. Serum 25(OH)D concentration values were available for 99% of the BWHS participants who provided blood samples.

In 2018, participants who lived in the Atlanta, GA area and had provided a blood sample in 2013 or 2014 were invited to provide a second blood sample. As a result, 510 women who provided a second blood sample had measured 25(OH)D available at two time points. These samples were used to assess within-person variability of plasma vitamin D over 4–5 years.

### Covariates

Data on individual-level covariates (age, weight, cigarette smoking, educational status, geographic region) were taken from the BWHS questionnaire closest in time and prior to the date a blood sample was provided; height was taken from the baseline questionnaire in 1995. If a variable was missing on that questionnaire, its value was taken from the most recently completed questionnaire before that. A neighborhood socioeconomic status (SES) score was derived by linking participants’ 2013 addresses to U.S. Census block group data on wealth, income, and education [29]. Data on household size and working outside the home even though it felt unsafe were taken from the BWHS COVID-19 supplemental questionnaire.

### Data analysis

**Fig 1:**
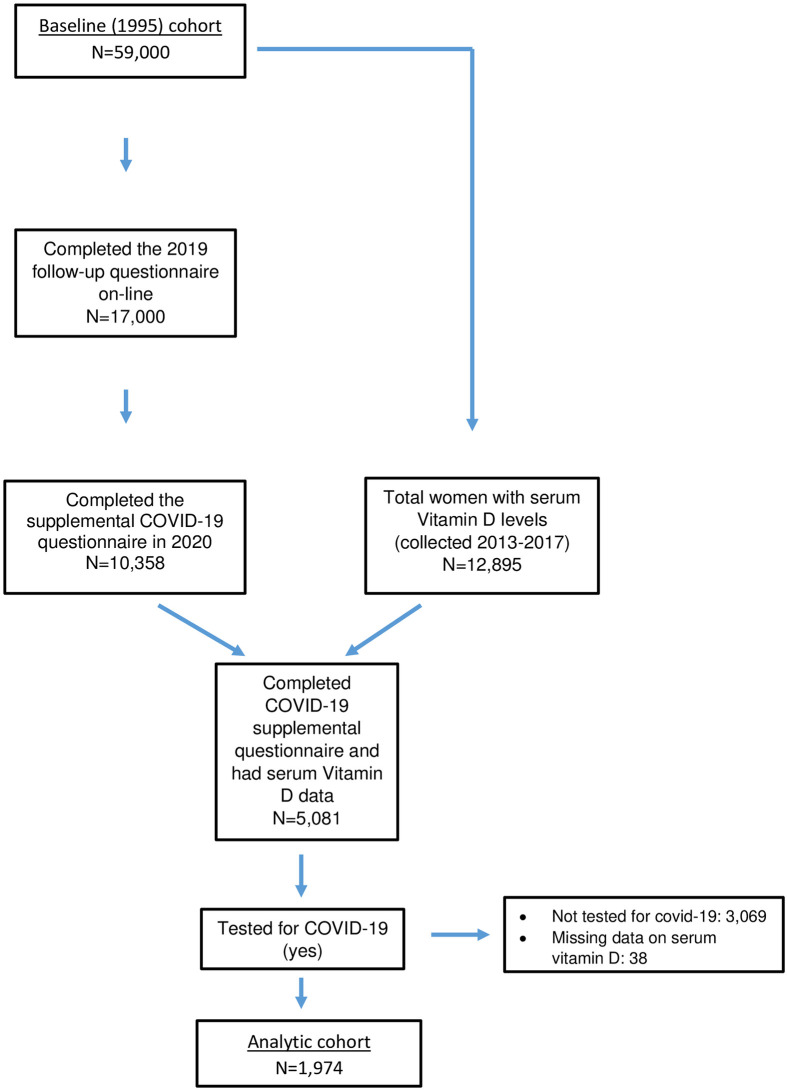
Black Women’s Health Study follow up flow chart, 1995–2020.

Among the 10,358 BWHS participants who completed the BWHS COVID-19 supplemental questionnaire, 5,081 had previously provided a blood sample. From among those 5,081 women, we excluded women who indicated they had not been tested for COVID-19 (n = 3,051), and those with missing COVID-19 test data (n = 56); the analytic sample was restricted to the 1,974 women who reported having had a COVID-19 test (Fig 1). All blood samples were obtained at least two years before potential exposure to COVID-19; the median interval was 5 years, range 3–7 years. Women in the analytic sample and those not included in the sample because they had not reported a COVID-19 test were generally similar: mean age, 62.9 and 62.7 years, respectively; mean BMI (body mass index, weight divided by height^2^ (kg/m^2^)), 30.8 and 30.7; at least 16 years of education, 72% and 66%; ever smoked, 34% and 31%.

Sample characteristics were compared across levels of 25(OH)D concentration by calculating analysis of variance (ANOVA) and chi-squared tests of proportions. Logistic regression analysis was used to estimate odds ratios (OR) and 95% confidence intervals (CI) for the association of 25(OH)D level with COVID-19 positivity among the 1,974 women tested, with a reference category of ≥30 ng/mL (≥75 nmol/l). 25(OH)D level was categorized according to the commonly used cut-points of <20, 20–29, and ≥30 ng/mL (<50, 50–72.5, and (≥75 nmol/l) (often referred to as deficient, insufficient, and sufficient, respectively) [30, 31]. We present results from both age-adjusted and multivariable models. The multivariable models include terms for age (continuous), BMI (continuous), geographic region (Northeast, South, Midwest, West), neighborhood socioeconomic status (quintiles), years of education (<16, ≥16 years), cigarette smoking (current, past, never), season of blood draw (winter/spring, summer/fall), number of individuals in household (1, 2, ≥3), and response to question on working outside the home even though it felt unsafe (yes/no). Tests for trend across categories of serum 25(OH)D were performed by entering the median value within each category. In addition, we performed analyses within strata of age (<63 (median age), ≥63 years), body mass index (<30, ≥30 kg/m^2^), and neighborhood SES score (below median, above median).

We conducted a sub-analysis of “severe” or “long-haul” COVID-infection restricted to the 41 women who reported that they had been hospitalized for COVID-19 or that their health had not returned to the usual level it had been before they had become infected.

Finally, we evaluated the reproducibility of 25(OH)D measurements over 4–5 years among 510 women with two blood measures by calculating an intra-class correlation coefficient (ICC) and 95% confidence interval (CI). The ICC measures the fraction of total variation due to between-person variability by dividing the between-person variance by the sum of the within- and between-person variances [32]. Using a mixed model, we adjusted for age (continuous) by including it as a fixed effect [33].

## Results

As shown in Table 1, 25(OH)D concentrations were higher in women who were older, thinner, never smoked, had a higher level of education, lived in the highest SES neighborhoods, and provided a blood sample during the summer months.

**Table 1:** Participant characteristics in relation to levels of serum 25(OH)D at the time of blood draw, Black Women’s Health Study.

	25(OH)D Concentration			*P* value
	< 20 ng/mL	20 – 29ng/mL	≥ 30ng/mL	
	(<50 nmol/l)	(50–72.5 nmol/l)	(75 nmol/l)	
**Characteristic**	(n = 373)	(n = 512)	(n = 1089)	
Age, years, mean (SD)	60.5 (8.4)	61.8 (8.4)	64.3 (8.5)	< 0.0001
Body mass index, kg/m2, mean (SD)	32.1 (7.1)	30.9 (6.7)	30.2 (6.8)	< 0.0001
Education in years, N (%)				0.0674
≤ 12	24 (7)	33 (7)	69 (6)	
13–15	103 (27)	101 (20)	233 (22)	
≥ 16	246 (66)	378 (74)	787 (72)	
Smoking status, N (%)				0.0032
Current	33 (9)	24 (5)	45 (4)	
Past	105 (31)	136 (28)	336 (29)	
Never	235 (60)	352 (67)	708 (67)	
Neighborhood socioeconomic score, N %)				0.0071
Lowest quintile	88 (22)	99 (20)	180 (16)	
Highest quintile	59 (18)	89 (17)	219 (20)	
Geographic region, N (%)				0.0435
Northeast	94 (25)	126 (24)	298 (28)	
South	110 (29)	193 (37)	404 (37)	
Midwest	98 (27)	116 (24)	242 (22)	
West	67 (19)	76 (15)	143 (13)	
Season of blood draw, N (%)				0.0145
Winter	99 (28)	108 (21)	271 (25)	
Spring	116 (30)	159 (31)	281 (26)	
Summer	67 (17)	133 (26)	273 (25)	
Fall	97 (25)	112 (22)	264 (24)	

**Percentages are age-standardized

Results from age-adjusted and multivariable models were similar (Table 2). Relative to women with 25(OH)D levels of 30 ng/mL (75 nmol/l) or more, age-adjusted ORs for COVID infection in women with levels of 20–29 ng/mL (50–72.5 nmol/l) and <20 ng/mL (<50 nmol/l) were 1.44 (95% CI 0.94–2.22) and 1.73 (95% CI 1.10–2.72), respectively, with p trend 0.01. Multivariable ORs for the same comparisons were 1.48 (95% CI 0.95–2.30) and 1.69 (95% CI 1.04–2.73), respectively, with p trend 0.02.

**Table 2:** Serum 25(OH)D concentrations in relation to COVID-19 infection, among 1,974 Black Women’s Health Study participants who provided a blood sample 3–7 years prior to 2020.

Serum 25(OH)D ng/mL (nmol/l)	Tested positive	Total tested	Age-adjusted odds ratio (95% CI)	Multivariable odds ratio* (95% C)
< 20 (<50)	33	373	1.73 (1.10–2.72)	1.69 (1.04–2.73)
20–29 (50–72.5)	38	512	1.44 (0.94–2.22)	1.48 (0.95–2.30)
≥ 30 (≥75)	56	1089	Reference	Reference
*p* trend			0.01	0.02

* Adjusted for age, body mass index, geographic region, neighborhood SES score, years of education, cigarette smoking, season of blood draw, household size, and working conditions during the pandemic

In multivariable models, COVID-19 risk factors of having ≥3 people in household (versus one person) and report of unsafe working conditions (versus report of not unsafe) were significantly associated with increased odds of testing positive (OR 2.14 (95% CI 1.36–3.38) and 1.77 (95% CI 1.08–2.89), respectively) (data not shown).

Results of stratified analyses are shown in Table 3. Associations in the overall data were consistent across strata of age and neighborhood SES. A stronger association of low 25(OH)D level with COVID-19 test positivity was observed among women with obesity (p trend 0.004), with little evidence of an association among leaner women (*P*_interaction_ with obesity status = 0.06).

**Table 3:** Serum 25(OH)D concentration in relation to COVID-19 infection, within strata of age, body mass index, and neighborhood socioeconomic score, Black Women’s Health Study.

Serum 25(OH)D concentration ng/mL (nmol/l)	Positive test/total	Age-adjusted odds ratio (95% CI)	Positive test/total	Age-adjusted odds ratio (95% CI)	*P*_*interaction*_
	Age				
	< 63 years		≥ 63 years		
< 20 (<50)	21 / 224	1.94 (1.04–3.60)	12 /149	1.53 (0.77–3.06)	0.61
20–29 (50–72.5)	22 / 270	1.67 (0.91–3.07)	16 / 242	1.24 (0.67–2.31)	0.50
≥ 30 (≥ 75)	23 / 465	Reference	33 / 624	Reference	
*p* trend		0.03		0.21	
	Body mass index				
	< 30 kg/m^2^		≥ 30 Kg/m^2^		
< 20 (<50)	11 / 159	1.13 (0.56–2.28)	22 / 214	2.65 (1.38–5.12)	0.06
20–29 (50–72.5)	21 / 255	1.37 (0.78–2.40)	17 / 257	1.70 (0.86–3.37)	0.57
≥ 30 (≥ 75)	38 / 623	Reference	18 / 466	Reference	
*p* trend		0.48		0.004	
	Neighborhood socioeconomic score				
	Below median		Above median		
< 20 (<50)	15 / 182	2.01 (0.99–4.08)	14 / 159	1.51 (0.78–2.95)	0.57
20–29 (50–72.5)	24 / 249	2.46 (1.33–4.55)	11 / 221	0.82 (0.40–1.68)	0.02
≥ 30 (≥ 75)	20 / 487	Reference	31 / 539	Reference	
*p* trend		0.02		0.34	

In a sub-analysis aimed at assessing more severe or long-haul COVID -19, we confined the outcome group to the 41 women who reported that they had been hospitalized for COVID-19 or that their health had not returned to the usual level it had been before they had become infected: the multivariable ORs the new outcome group were 1.13 (95% CI 0.50–2.57) and 2.24 (95% CI 1.05–4.76),(p trend 0.05), for women with 25(OH)D levels of 20–29 ng/mL and <20 ng/mL (50–72.5 nmol/l and <50 nmol/l) respectively relative to levels of ≥ 30 ng/m (≥75 nmol/l).

Finally, we assessed the reproducibility of serum 25(OH)D in participants over a period of 4–5 years among the 510 participants who provided a second blood sample in 2018. The ICC was 0.50 (95% CI: 0.43, 0.56) indicating moderate reproducibility [34].

## Discussion

The current analysis is the first, to our knowledge, to examine the relation of 25(OH)D level to COVID-19 infection in Black women, a population disproportionately impacted by the current pandemic. Importantly, we were able to adjust for a number of factors related to risk of becoming infected with COVID-19, including number of people in the household, years of education (a marker for employment status and individual socioeconomic status), and socioeconomic characteristics of the residential neighborhood. We found that the odds of testing positive for COVID-19 increased with decreasing level of level of 25(OH)D, such that women with levels considered to be deficient (< 20 ng/mL; <50 nmol/l) were estimated to have a 69% greater odds of infection relative to women with a vitamin D level of 30 ng/mL (75 nmol/l) or greater. The association was primarily present among women with obesity.

Vitamin D is recognized to have a role in the regulation of the immune response. The vitamin D receptor (VDR) is present in almost all immune cells and has effects on CD4+ and CD8+ T-cells, B-cells, dendritic cells, and macrophages [20]. In response to invading pathogens, epithelial cells–the primary barrier between the environment and the body–activate antigen-presenting cells (macrophages, dendritic cells) to synthesize 1,25-dihydroxyvitamin D (1,25 (OH)_2_D) from its precursor 25(OH)D by means of the enzyme 1α-hydroxylase (CYP27B1) [35]. The impact of vitamin D on the immune response varies by cell type, but overall vitamin D induces a more immune-tolerant status [36]. Macrophages, for example, respond with increased interleukin (IL)-10 production and decreased inflammation [37]. Furthermore, 1,25(OH)_2_D promotes downregulation of pro-inflammatory Th1 lymphocytes and induction of regulatory T cells while also increasing expression of antimicrobial peptides (cathelicidin, β-defensin2). Concurrently, 1,25(OH)_2_D minimizes bystander damage from pathogens by reducing neutrophil migration. Thus, low serum 25(OH)D may impair immune function if less is available for synthesis of 1,25(OH)_2_D [38]. Additionally, vitamin D deficiency may potentially affect susceptibility to COVID-19 through its role in activating the pulmonary renin angiotensin system and regulating ACE2 which plays a role in lung injury protection [39].

In the largest previous study, Kaufman and colleagues [12] found a strong inverse relationship between circulating 25(OH)D levels and COVID-19 test positivity rates in data based on specimens analyzed at a large national clinical laboratory, but information on patient race had not been collected. The investigators used patient zip codes to classify individuals as to race/ethnicity based on the race/ethnicity proportions reported in the 2018 American Community Survey. Zip codes with an estimated proportion of Black residents of at least 50% were designated “predominantly Black non-Hispanic”. Among those who resided in “predominantly Black non-Hispanic” zip codes (about 10% of the study population), there was an inverse association of 25(OH)D level with test positivity, consistent with the overall findings. The investigators were not able to assess the role of confounding by other COVID-19 risk factors, such as living in crowded conditions and working at high-risk jobs, which are also more common among individuals who live in predominantly Black neighborhoods.

A small hospital-based study (N = 107) in Switzerland, assessed patients who were tested for both COVID-19 and 25(OH)D during 2020. Compared to patients negative for COVID-19, positive patients had significantly lower 25(OH)D levels (p = 0.004) [13]. A study conducted at a U.S. urban medical center assessed 489 patients, including 286 Blacks, whose vitamin D levels were measured within the 12 months prior to being tested for COVID-19. Compared to those whose 25(OH)D level was ≥ 20 ng/mL (≥50 nmol/l), those classified as deficient (< 20 ng/mL; <50 nmol/l) had a 77% increased risk of testing positive for COVID-19 (*p* = 0.02) [14]. Results were not reported separately by race/ethnicity. By contrast, a study based on UK Biobank data from 348,598 individuals, of whom 449 had confirmed COVID-19 infections, found that the median 25(OH)D) concentrations measured at study recruitment (2006–2010) were similar for individuals who tested positive for COVID-19 infection and those who tested negative for COVID-19 in 2020 (OR = 1.00, p = 0.21) [16]. It is possible that the long delay between vitamin D measurement and COVID infection affected the study findings.

We assessed 25(OH)D and COVID-19 infection within several subgroups. The association of 25(OH)D concentration with COVID-19 was markedly stronger among obese women. This finding is important because the prevalence of obesity is disproportionately high in US Black women [40–42], with a prevalence of 49.6%. It is clear that obesity is associated with increased odds of severe COVID and COVID-related death [43]. Whether obesity may also enhance risk of infection is unknown, but such an effect has been reported for other viral infections, such as the H1N1 influenza [44]. In terms of mechanisms, excess body fat and obesity is inversely associated with 25(OH)D [45] and may contribute to immunologic deficits and inability to mount an effective host defense [43, 46]. If women with obesity have less resistance to infection to begin with, it is possible that 25(OH)D deficiency, with its own biologic consequences, could have a synergistic effect, resulting in the higher odds of COVID infection observed among women with obesity and 25(OH)D deficiency.

In the present study, 25(OH)D levels were obtained from samples provided 3–7 years before COVID-19 infection and may not be an accurate representation of serum concentrations at the time of COVID-19 infection. This may have led to non-differential misclassification of 25(OH)D levels, which could result in an underestimation of the magnitude of the association, but would not have produced an association where none exists. However, it may be that 25(OH)D levels in individuals do not change enough over time to move an appreciable number of individuals from one category to another category of the three levels of 25(OH)D considered in the analysis. Kotsopoulos and colleagues examined the 2–3 year reproducibility of plasma 25(OH)D among 40 premenopausal and 75 postmenopausal women enrolled in the Nurses’ Health Study (NHS) and the Nurses’ Health Study II (NHSII). They estimated an ICC of 0.75 indicating good reproducibility [47]. A later study by Bertrand et al., conducted among 443 women in the NHS with two blood samples taken 10–11 years apart estimated an ICC of 0.50 indicating moderate reproducibility [48]. Our own analyses involving 510 women who provided two blood samples 4–5 years apart resulted in an ICC of 0.50, an indication of moderate reproducibility of serum 25(OH)D levels. Misclassification of long-term serum 25(OH)D concentration, if random, would have biased our estimates towards the null.

We relied on self-report of COVID-19 testing status, possibly leading to non-differential misclassification of outcome and underestimation of the magnitude of association. We have previously found high validity of self-report for numerous health conditions in the BWHS including type 2 diabetes [49], hypertension [50], and sarcoidosis [51], and it is highly likely that participants accurately recall their COVID infection status given the potential consequences of infection. However, it is estimated that approximately 40–45% of COVID-19 infection is asymptomatic [52]. Thus, asymptomatic infection was likely underdiagnosed in our study. Unless detection of asymptomatic disease was somehow related to 25(OHD levels, the result of underdiagnosis of asymptomatic disease would have underestimated the association between 25(OH)D level and COVID-19 infection.

A key strength of the study was availability of data on socioeconomic status, as measured by educational status and neighborhood characteristics. These factors are potential confounders of any analysis related to COVID-19 infection because individuals with low educational levels and those who live in disadvantaged neighborhoods are more likely to have been exposed to the virus. Data were also available on number of people in the household and on whether the participants considered their work outside the home to be safe or unsafe with regard to exposure to COVID-19, and these factors were also included in multivariable models.

However, we had no information on current occupation or on use of PPEs and thus were unable to adjust for these factors. Serum concentrations of 25(OH)D in BWHS participants were measured at a certified clinical laboratory under standard conditions. Finally, women who reported being tested for COVID-19 were similar with regard to BMI and other characteristics to those who had given blood samples but who reported not being tested for COVID, suggesting that the women who reported testing were not a biased sample.

## Conclusion

In conclusion, we found an inverse association between serum 25(OH)D concentration and risk of COVID-19 test positivity in a national cohort of U.S. Black women, particularly among obese women. Importantly, by carrying out the analysis in a study restricted to Black women, we lessened concerns about uncontrolled confounding due to the higher levels of COVID-19 exposure experienced by Black Americans. Our data add to the body of literature supporting a potential role for 25(OH)D in relation to risk of COVID-19 infection, and are of particular importance to U.S. Blacks, who generally have lower levels of 25(OH)D. Further study is needed to confirm these findings and determine what level of 25(OH)D is optimal. In addition, future studies exploring endpoints including severe COVID-19 –hospitalization and death–are warranted.
